# Predictors of hypocalcaemia and hypoparathyroidism in patients undergoing thyroidectomy for benign and malignant pathologies

**DOI:** 10.1530/EO-24-0022

**Published:** 2024-11-25

**Authors:** Jino Johns Lalitha, Natarajan Ramalingam, Remya Rajan, Jeyashanth Riju, Antony Abraham Paulose, Rajiv Charles Michael, Amit Jiwan Tirkey, Twisha Adhikari, Shalini Sahu, R Nagayazhini

**Affiliations:** 1 Department of Head & Neck Surgery, Christian Medical College, Vellore, India; 2 Department of Endocrinology, Christian Medical College, Vellore, India; 3 Department of General Pathology, Christian Medical College, Vellore, India; 4 Department of Radiology, Christian Medical College, Vellore, India; 5 Department of Biostatistics, Christian Medical College, Vellore, India

**Keywords:** thyroid neoplasm, thyroidectomy, hypocalcaemia, hypoparathyroidism, neck dissection

## Abstract

**Objective:**

To analyse the risk factors of hypoparathyroidism and hypocalcaemia after total thyroidectomy.

**Methods:**

Clinical data of patients who underwent total thyroidectomy at a tertiary care hospital in southern part of India were collected retrospectively from January 2021 to May 2023. Multivariate logistic regression was used to analyse the risk factors associated with transient hypoparathyroidism and hypocalcaemia separately.

**Results:**

A total of 300 patients who underwent total thyroidectomy were enroled. The median age of the study population was 41 years, and 70% were females. Histopathological examination showed that 80.3% had thyroid cancer. The incidence of postoperative transient hypoparathyroidism was 26.7%, while postoperative transient hypocalcaemia was 12.3%. Multivariate analysis showed that the presence of hypothyroidism before surgery (OR = 3.230, 95% CI: 1.368–7.624, *P* = 0.007), performing central compartment neck dissection (CCND) (OR = 2.196, 95% CI: 1.133–4.257, *P* = 0.02) and parathyroid gland in the surgical specimen (OR = 5.547, 95% CI: 3.065–10.036, *P* < 0.0001) were independent predictors of postoperative transient hypoparathyroidism. Female gender (OR = 2.689, 95% CI: 1.049–6.895, *P* = 0.039), presence of parathyroid in the surgical specimen (OR = 1.067, 95% CI: 0.367–8.438, *P* = 0.004) and performing CCND (OR = 2.192, 95% CI: 0.990–4.850, *P* = 0.05) were independent predictors of postoperative transient hypocalcaemia.

**Conclusion:**

Hypoparathyroidism and hypocalcaemia after thyroid surgery are common, and most of them are transient. The independent predictors of hypoparathyroidism and hypocalcaemia differ. Hypoparathyroidism appears to be a better predictor for patients who will develop postoperative hypocalcaemia.

## Introduction

With the growing incidence of thyroid malignancy and improved detection of thyroid nodules using diagnostic imaging, the number of patients requiring thyroid surgery has increased (Kitahara & Sosa 2016). The most common complication following total thyroidectomy (TT) is hypoparathyroidism and the resultant hypocalcaemia. Despite advances and improvements in surgical techniques, hypoparathyroidism is still an unresolved problem (Promberger *et al.,* 2014). Direct injury to, or devascularisation of, the parathyroid glands is the most common cause of post-thyroidectomy hypocalcaemia (Edafe *et al.,* 2014). Measuring serum parathormone (PTH) post-thyroidectomy is a sensitive method for identifying patients at risk for hypocalcaemia. PTH is the main factor that regulates calcium, and small changes in PTH levels alter calcium levels.

Transient or temporary hypoparathyroidism is defined as hypoparathyroidism lasting less than 6 months after surgery, while permanent hypoparathyroidism persists beyond 6 months after surgery (Shoback *et al.* 2016). The incidence of transient hypoparathyroidism in the literature ranges from 14% to 60% (Nagel *et al.* 2022), and the median incidence is 27% (interquartile range = 19–38) as per the meta-analysis by Edafe *et al.* (2014). The incidence of permanent hypoparathyroidism ranges from 0% to 4% (Edafe *et al.* 2014, Nagel *et al.* 2022). Transient or temporary hypoparathyroidism can be defined as an undetectable or inappropriately low postoperative PTH level (<10 pg/mL) in the context of hypocalcaemia, with or without hypocalcaemia symptoms (Nagel *et al.* 2022). Transient hypocalcaemia is defined as a calcium level below the lower limit of normal, i.e. 2.0 mmol/L or less than 8.0 mg/dL (Edafe *et al.* 2014, Nagel *et al.* 2022). Studies have reported various risk factors as predictors of hypocalcaemia following TT, and the results have been inconsistent across studies (Erbil *et al.* 2009, Edafe *et al.* 2014). Therefore, accurate judgment and identification of risk factors for postoperative hypoparathyroidism are necessary to reduce the morbidity of hypocalcaemia and would help to initiate early treatment following surgery.

This study aims to investigate the prevalence and risk factors for post-thyroidectomy transient hypoparathyroidism and hypocalcaemia following TT in the Indian setting.

## Materials and methods

We performed a retrospective analysis of patients who underwent TT or completion thyroidectomy surgery in the Department of Head and Neck Surgery at a tertiary care hospital in the southern part of India between January 2021 and May 2023. This study obtained ethical approval from the Institutional Review Board (IRB min no. 15756(retro), dated 20.09.2023). A total of 300 consecutive patients who underwent TT in the Department of Head and Neck Surgery were included. Patients with renal failure, concomitant parathyroid disorders, recurrent thyroid swelling following TT and those with a prior history of radiation therapy to the head and neck region were excluded.

**Figure 1:**
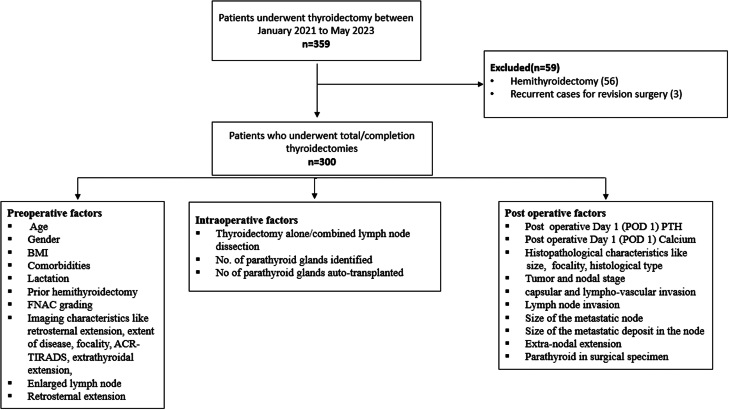
Study flowchart.

The serum calcium and PTH levels of patients were tested postoperatively on day 1 and further as per clinical indication. The estimation of serum calcium was performed using the photometric method (Cobas^®^ C 702 analyser (Roche)), and intact PTH was estimated by chemiluminescence (Atellica^®^ IM Intact PTH assay – Siemens). Patients were considered to have transient hypoparathyroidism if the postoperative day 1 (POD 1) PTH value was <10 pg/mL and transient hypocalcaemia if the POD 1 serum calcium value was <8 mg/dL (Nagel *et al.* 2022). Patients were started on treatment based on department protocol. Pre-operative, intraoperative, and postoperative characteristics (Fig. 1) were collected for the study subjects from electronic medical records. The study flow diagram and parameters considered are shown in Fig. 1. Patients who were on thyroxine replacement preoperatively, or those who were diagnosed to have hypothyroid during evaluation for thyroid nodule, were considered as hypothyroid. All these patients underwent medical management before surgery and were operated on in an euthyroid state.

The statistical analysis was performed using IBM SPSS Statistics for Windows, Version 20 (IBM Corp.) and R (version 4.3.1). The analysis of the relationship between categorical variables was performed using contingency tables and the chi-square test/Fisher test. Spearman’s rank order correlation was used to assess the relationship between postoperative hypocalcaemia and postoperative PTH. Odds ratios (OR) were reported with a 95% CI and *P* values. The level of statistical significance was determined at *P* ≤ 0.05. Predictive factors of hypoparathyroidism and hypocalcaemia were analysed separately. A *P*-value ≤0.05 in univariate analysis was considered for the multivariate model, using a downward stepwise binary logistic regression analysis.

## Results

A total of 300 patients who underwent thyroid surgery (Fig. 1) were included in the study. The study included 210 women and 90 men, with a median age of 41.5 years. Table 1 lists the baseline demographic and clinicopathological characteristics of the enroled patients. The final histopathological examination showed 80.3% of the patients had malignancy. Out of this, 77% (*n* = 231) had papillary thyroid carcinoma, and rest included Hürthle cell carcinoma (0.7%), high-grade differentiated thyroid cancer (0.7%), poorly differentiated thyroid cancer (1%), medullary thyroid carcinoma (0.7%) and anaplastic thyroid cancer (0.3%). Central compartment neck dissection (CCND) was performed in 19.7% of the cases. The presence of thyroiditis was histologically confirmed in 36.3% of cases. The mean number of parathyroid glands identified during surgery was 3.69 (s.d. 0.654). Inadvertent removal of parathyroid glands, during surgery, as confirmed by histopathology, was noted in 26.3% of patients.

**Table 1:** Demographic and clinicopathological data.

Patient characteristic	*n* (%)
Gender	
Female Male	210 (70) 90 (30)
BMI (median)	24.1 ± 3.9
Surgical procedure	
Thyroidectomy alone Thyroidectomy + central/lateral neck dissection	240 (80) 60 (20)
Histology	
Benign Malignant	59 (19.7) 241 (80.3)
No. of parathyroid glands identified	
One Two Three Four	5 (1.7) 52 (17.3) 38 (12.7) 205 (68.3)
No. of parathyroid auto transplanted	9 (3)
No. of patients with parathyroid glands observed in surgical specimen	79 (26.3)
Postoperative temporary hypoparathyroidism	80 (26.7)
Postoperative temporary hypocalcaemia	37 (12.3)
Postoperative permanent hypoparathyroidism	7 (2.3)
Mean size of largest nodule on USG	3.9 cm ± 2.2
FNAC – Bethesda grading (*n* = 280, missing = 20)	
Bethesda 1 Bethesda 2 Bethesda 3–4 Bethesda 5–6	56 (18.7) 45 (15) 100 (35.7) 79 (28.2)
Histopathological parameters (*n* = 300)	
Thyroiditis	109 (36.3)
pT stage (*n* = 241, missing = 59)	
pT1–2 pT3–4	158 (65.2) 83 (35)
pN stage (*n* = 78, missing = 222)	
pN1a pN1b	33 (42.3) 45 (57.7)

BMI, body mass index; CCND, central compartment neck dissection; FNAC, fine needle aspiration cytology; TT, total thyroidectomy; p, pathological.

**Figure 2:**
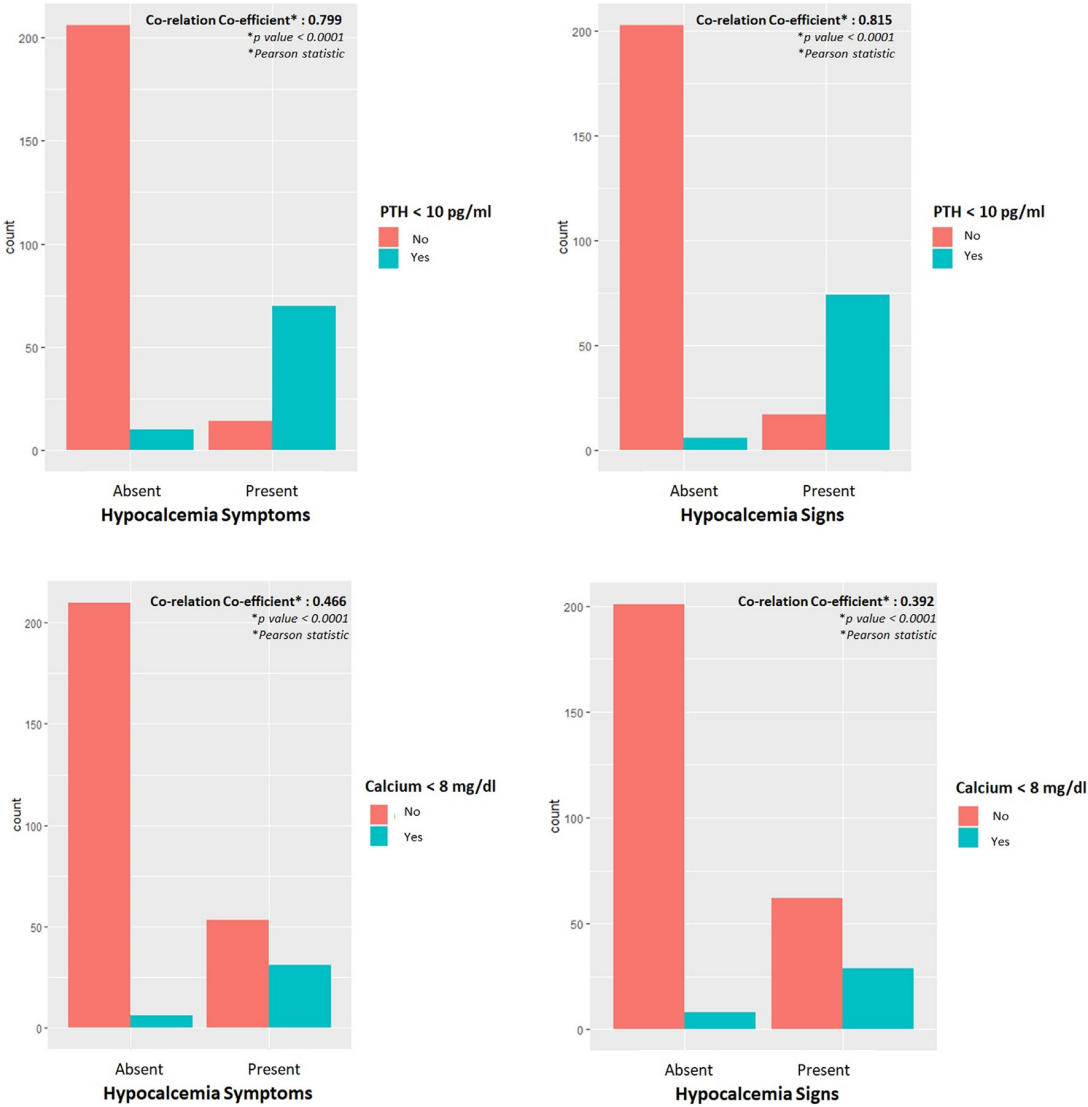
Co-relation of clinical features with postoperative day 1 PTH and calcium values.

**Figure 3:**
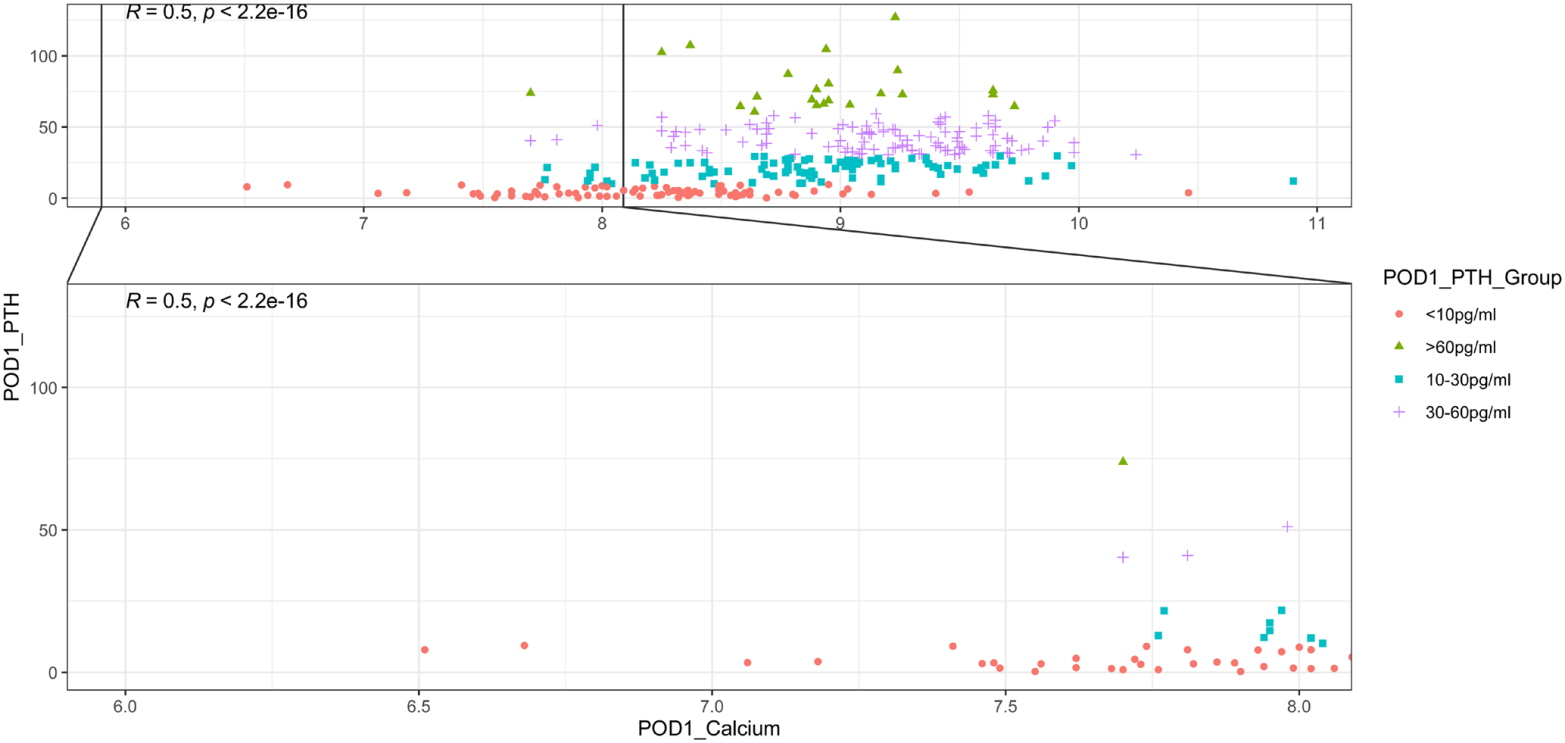
Co-relation between postoperative day 1 PTH and calcium values.

Although 80 (26.7%) patients had biochemical postoperative hypoparathyroidism on POD1, hypocalcaemia was present in only 37 (12.3%) patients. Following surgery, 28% (*n* = 84) of patients had symptoms of hypocalcaemia, and 30.3% (*n* = 91) had signs of hypocalcaemia. Six patients with symptoms had no demonstrable clinical sign of hypocalcaemia, while 13 patients without any symptoms had an elicitable clinical signs of hypocalcaemia. We found that serum PTH levels had a better coefficient of correlation than serum calcium levels with both patient-reported symptoms and clinically elicited signs of hypocalcaemia (Fig. 2). POD1 PTH values correlated better with hypocalcaemia symptoms (*r* = 0.799 vs 0.466) and signs (*r* = 0.815 vs 0.392) compared to calcium levels (Fig. 2). There was a positive correlation between POD1 calcium and PTH (*r* = 0.5, *P*-value = 2.2e–16) (Fig. 3). Of all patients who had a calcium level of <8 mg/dL (*n* = 37), 72.9% (*n* = 27) of patients had a PTH value of less than 10 pg/mL.

Of the patients who had hypothyroidism preoperatively (*n* = 32), 40.6% developed transient hypoparathyroidism. The presence of a significant central compartment neck node (CCN) on radiological examination could predict hypocalcaemia in 56.2%. Table 2 lists the univariate analysis for predictors of transient hypoparathyroidism. Among these, seven factors were significantly related to the presence of postoperative hypoparathyroidism, namely, the presence of hypothyroidism (OR = 2.460, 95% CI: 1.123–5.387, *P* = 0.024), surgery type (OR = 2.634, 95% CI: 1.45–4.76, *P* = 0.001), CCND (OR = 2.487, 95% CI: 1.36–4.52, *P* = 0.003), presence of parathyroid gland in the surgical specimen (OR = 5.467, 95% CI: 3.11–9.60, *P = *<0.0001), radiological CCN in imaging (OR = 3.857, 95% CI: 1.38–10.73, *P* = 0.010), presence of pathological positive node (OR = 2.788, 95% CI: 1.06–7.30, *P* = 0.037) and metastatic node size more than 3 cm (OR = 5.5, 95% CI: 1.56–19.36, *P* = 0.008). CCN, presence of pathological positive node and node >3 cm were not included in multivariate analysis because of low column counts (*n* < 10) and wide CIs.

**Table 2:** Univariate analysis for factors predicting transient hypoparathyroidism^a^.

Variable	Postop. Day 1 PTH Level (*n* = 300)		Odds Ratio (95% CI)	*P*
	>10 pg/mL	<10 pg/mL		
Age				
<40 years >40 years	100 (45.5%) 120 (54.5%)	40 (50.0%) 40 (50.0%)	0.833 (0.49–1.39)	0.486
Gender				
Female Male	148 (67.3%) 72 (32.7%)	62 (77.5%) 18 (22.5%)	0.597 (0.32–1.08)	0.089
Body mass index (BMI)				
<24.99 kg/m^2^ >25 kg/m^2^	126 (57.3%) 94 (42.7%)	51 (63.8%) 29 (36.2%)	0.762 (0.44–1.29)	0.314
Comorbidity				
No Yes Hypothyroidism	151 (68.6%) 50 (22.7%) 19 (8.6%)	42 (52.5%) 25 (31.2%) 13 (16.2%)	Ref 1.798 (0.997–3.241) 2.460 (1.123–5.387)	– 0.051 **0.024**
Surgery type				
TT TT + ND	186 (84.5%) 34 (15.5%)	54 (67.5%) 26 (32.5%)	2.634 (1.45–4.76)	**0.001**
CCND				
No Yes	186 (84.5%) 34 (15.5%)	55 (68.8%) 25 (31.2%)	2.487 (1.36–4.52)	**0.003**
Histology				
Benign Malignant	43 (19.5%) 177 (80.5%)	16 (20.0%) 64 (80.0%)	0.930 (0.51–1.84)	0.972
Presence of parathyroid glands in surgical specimen				
No Yes	183 (83.2%) 37 (16.8%)	38 (47.5%) 42 (52.5%)	5.467 (3.11–9.60)	**<0.0001**
Radiological extrathyroidal extension				
No Yes	203 (92.3%) 17 (7.7%)	73 (91.2%) 7 (8.8%)	1.145 (0.45–2.87)	0.773
Radiological central compartment node				
No Yes	213 (96.8%) 7 (3.2%)	71 (88.8%) 9 (11.2%)	3.857 (1.38–10.73)	**0.010**
Radiological lateral compartment node				
No Yes	166 (75.5%) 54 (24.5%)	53 (66.2%) 27 (33.8%)	1.566 (0.89–2.73)	0.114
Radiological retrosternal extension				
No Yes	206 (93.6%) 14 (6.4%)	79 (98.8%) 1 (1.2%)	0.186 (0.02–1.44)	0.107
ACR TIRADS (*n* = 268, missing = 32)				
TIRADS 1 TIRADS 2 TIRADS 3 TIRADS 4 TIRADS 5	4 (2.1%) 13 (6.7%) 58 (29.7%) 74 (37.9%) 46 (23.6%)	1 (1.4%) 2 (2.7%) 24 (32.9%) 30 (41.1%) 16 (21.9%)	Ref 0.61 (0.04–8.70) 1.655 (0.17–15.58) 1.622 (0.17–15.11) 1.391 (0.14–13.38)	– 0.719 0.660 0.671 0.775
Focality (*n* = 284, missing = 16)				
Solitary Multifocal	68 (32.9) 139 (67.1)	23 (29.9) 54 (70.1)	1.149 (0.65–2.02)	0.632
FNAC (*n* = 280, missing = 20)				
Bethesda 1 Bethesda 2 Bethesda 3 Bethesda 4 Bethesda 5 Bethesda 6	41 (20.1%) 33 (16.2%) 70 (34.3%) 8 (3.9%) 24 (11.8%) 28 (13.7%)	15 (19.7%) 12 (15.8%) 20 (26.3%) 2 (2.6%) 13 (17.1%) 14 (18.4%)	Ref 0.994 (0.40–2.41) 0.781 (0.36–1.69) 0.683 (0.13–3.58) 1.481 (0.60–3.63) 1.367 (0.57–3.27)	– 0.989 0.530 0.653 0.391 0.483
Thyroiditis				
No Yes	145 (65.9%) 75 (34.1%)	46 (57.5%) 34 (42.5%)	1.429 (0.84–2.41)	0.182
Pathological T stage				
pT1a pT1b pT2 pT3a pT3b pT4a pT4b	37 (20.9%) 19 (10.7%) 60 (33.9%) 45 (25.4%) 13 (7.3%) 3 (1.7%) 0	13 (20.3%) 7 (10.9%) 22 (34.4%) 13 (20.3%) 7 (10.9%) 1 (1.6%) 1 (1.6%)	Ref 1.049 (0.35–3.06) 1.044 (0.47–2.32) 0.822 (0.34–1.98) 1.533 (0.50–4.67) 0.949 (0.09–9.94) –	– 0.931 0.917 0.664 0.453 0.965 –
Pathological focality				
Single Multifocal	72 (40.7%) 105 (59.3%)	20 (31.2%) 44 (67.8%)	1.509 (0.82–2.77)	0.185
Pathological extrathyroidal extension				
No Yes	161 (91.0%) 16 (9.0%)	55 (85.9%) 9 (14.1%)	1.647 (0.68–3.93)	0.262
Capsular invasion				
No Yes	152 (85.9%) 25 (14.1%)	56 (87.5%) 8 (12.5%)	0.869 (0.37–2.03)	0.746
Lympho-vascular invasion				
No Yes	117 (66.1%) 60 (33.9%)	35 (54.7%) 29 (45.3%)	1.616 (0.90–2.89)	0.106
Pathological N stage (*n* = 78, missing = 222)				
pN1a pN1b	24 (52.2%) 22 (47.8%)	9 (28.1%) 23 (71.9%)	2.788 (1.06–7.30)	**0.037**
Node size (*n* = 78, missing = 222)				
<3 cm >3 cm	42 (91.3%) 4 (8.7%)	21 (65.6%) 11 (34.4%)	5.5 (1.56–19.36)	**0.008**
Size of nodal metastatic foci (*n* = 78, missing = 222)				
<0.2 cm >0.2 cm	19 (31.3%) 27 (58.7%)	7 (21.9%) 25 (78.1%)	2.513 (0.90–6.99)	0.078
Pathological extra-nodal extension (*n* = 78, missing = 222)				
No Yes	35 (76.1%) 11 (23.9%)	25 (78.1%) 7 (21.9%)	0.891 (0.30–2.61)	0.834

^a^Binary logistic regression.

BMI, body mass index; CCND, central compartment neck dissection; FNAC, fine needle aspiration cytology; OR, odds ratio; TT, total thyroidectomy; TIRADS, thyroid imaging reporting and data systems.

For hypocalcaemia, univariate analysis (Table 3) showed that female gender (OR = 0.412, 95% CI: 0.166–1.026, *P* = 0.05), surgery type (OR = 2.150, 95% CI: 1.009–4.579, *P* = 0.047), CCND (OR = 2.206, 95% CI: 1.035–4.704, *P* = 0.041), parathyroid tissue in surgical specimen (OR = 3.137, 95% CI: 1.549–6.352, *P* = 0.001) and POD 1 PTH level less than 10 pg/mL (OR = 10.70, 95% CI: 4.89–23.47, *P* = <0.0001) were significant predictors of postoperative hypocalcaemia. Because of the significant correlation between POD 1 PTH and POD 1 calcium levels (Fig. 3), we did not consider that factor in multivariate analysis.

**Table 3:** Univariate analysis for factors predicting transient hypocalcaemia^a^.

Variable	Postop. Day 1 Calcium Level (*n* = 300)		Odds Ratio (95% CI)	*P*
	>8 mg/dL	<8 mg/dL		
Age				
<40 years >40 years	119 (45.2%) 144 (54.5%)	21 (56.8%) 16 (43.2%)	0.630 (0.314–1.261)	0.192
Gender				
Female Male	179 (68.1%) 84 (31.9%)	32 (83.8%) 6 (16.2%)	0.412 (0.166–1.026)	**0.057**
Body mass index (BMI)				
<24.99 kg/m*^2^* >25 kg/m^2^	152 (57.8%) 111 (42.2%)	25 (67.6%) 12 (32.4%)	0.657 (0.317–1.365)	0.260
Comorbidity				
No Cardiovascular Hypothyroidism	166 (63.1%) 70 (26.6%) 27 (10.3%)	27 (73.0%) 5 (13.5%) 5 (13.5%)	Ref 0.439 (0.162–1.187) 1.139 (0.404–3.212)	– 0.105 0.806
Surgery type				
TT TT + ND	215 (81.7%) 48 (18.3%)	25 (67.6%) 12 (32.4%)	2.150 (1.009–4.579)	**0.047**
CCND				
No Yes	216 (82.1%) 47 (17.9%)	25 (67.6%) 12 (32.4%)	2.206 (1.035–4.704)	**0.041**
Histology				
Benign Malignant	51 (19.4%) 212 (80.6%)	8 (21.6%) 29 (78.4%)	0.872 (0.376–2.021)	0.749
Presence of parathyroid glands in surgical specimen				
No Yes	202 (76.8%) 61 (23.2%)	19 (51.4%) 18 (48.6%)	3.137 (1.549–6.352)	**0.001**
Radiological extrathyroidal extension				
No Yes	247 (94.7%) 21 (8.0%)	36 (97.3%) 3 (8.1%)	1.017 (0.288–3.591)	0.979
Radiological central compartment node				
No Yes	251 (95.4%) 12 (4.6%)	30 (88.2%) 4 (10.8%)	2.535 (0.773–8.320)	0.125
Radiological lateral compartment node				
No Yes	195 (74.1%) 68 (25.9%)	24 (64.9%) 13 (35.1%)	1.553 (0.749–3.221)	0.237
Radiological retrosternal extension				
No Yes	249 (94.7%) 14 (5.3%)	36 (97.3%) 1 (2.7%)	0.494 (0.063–3.87)	0.502
ACR TIRADS (*n* = 268, missing = 32)				
TIRADS 1 TIRADS 2 TIRADS 3 TIRADS 4 TIRADS 5	4 (1.7%) 14 (6.0%) 71 (30.3%) 92 (39.3%) 53 (22.6%)	1 (2.9%) 1 (2.9%) 11 (32.4%) 12 (35.3%) 9 (26.5%)	Ref 0.286 (0.014–5.660) 0.620 (0.063–6.068) 0.522 (0.054–5.062) 0.679 (0.068–6.791)	– 0.411 0.681 0.575 0.742
Focality (*n* = 284, missing = 16)				
Solitary Multifocal	79 (31.6) 171 (68.4)	12 (35.3) 22 (64.7)	0.847 (0.399–1.797)	0.665
FNAC (*n* = 280, missing = 20)				
Bethesda 1 Bethesda 2 Bethesda 3 Bethesda 4 Bethesda 5 Bethesda 6	50 (20.5%) 41 (16.2%) 70 (34.3%) 8 (3.9%) 24 (11.7%) 33 (13.5%)	6 (16.7%) 4 (11.1%) 11 (30.6%) – 6 (16.7%) 9 (25.0%)	Ref 0.813 (0.215–3.077) 1.160 (0.404–3.335) – 1.613 (0.478–5.447) 2.273 (0.740–6.984)	– 0.760 1.160 – 0.441 0.152
Thyroiditis				
No Yes	171 (65.0%) 92 (35.0%)	20 (54.1%) 17 (45.9%)	1.580 (0.789–3.164)	0.197
Pathological T stage (*n* = 241, missing = 59)				
pT1a pT1b pT2 pT3a pT3b pT4a pT4b	45 (21.2%) 22 (10.4%) 73 (34.4%) 52 (24.5%) 16 (7.5%) 3 (1.4%) 1 (0.5)	5 (17.2%) 4 (13.8%) 9 (31.0%) 6 (20.7%) 4 (13.8%) 1 (3.4%) –	Ref 1.636 (0.399–6.704) 1.110 (0.350–3.520) 0.038 (0.297–3.632) 2.250 (0.537–9.432) 3.0 (0260–34.575) –	– 0.494 0.860 0.953 0.267 0.378 –
Pathological focality				
Single Multifocal	83 (39.2%) 129 (60.8%)	9 (31.0%) 20 (69.0%)	1.430 (0.621–3.291)	0.401
Pathological extrathyroidal extension				
No Yes	192 (90.6%) 20 (9.4%)	24 (82.8%) 5 (17.2%)	2.000 (0.687–5.819)	0.203
Capsular invasion				
No Yes	180 (84.9%) 32 (15.1%)	28 (96.6%) 1 (3.4%)	0.201 (0.026–1.529)	0.121
Lympho-vascular invasion				
No Yes	133 (62.7%) 79 (37.3%)	19 (65.5%) 10 (34.5%)	0.886 (0.392–2.001)	0.771
Pathological N stage (*n* = 78, missing = 222)				
pN1a pN1b	30 (44.8%) 37 (55.2%)	3 (27.3%) 8 (72.7%)	2.162 (0.527–8.870)	0.284
Node size (*n* = 78, missing = 222)				
<3 cm >3 cm	55 (82.1%) 12 (17.9%)	8 (72.7%) 3 (27.3%)	1.719 (0.397–7.449)	0.469
Size of nodal metastatic foci (*n* = 78, missing = 222)				
<0.2 cm >0.2 cm	24 (35.8%) 43 (64.2%)	2 (18.2%) 9 (81.8%)	2.51 (0.501–12.58)	0.263
Pathological extra-nodal extension (*n* = 78, missing = 222)				
No Yes	53 (79.1%) 14 (20.9%)	7 (63.6%) 4 (36.4%)	2.163 (0.554–8.448)	0.267

^a^Binary logistic regression.

BMI, body mass index; CCND, central compartment neck dissection; FNAC, fine needle aspiration cytology; OR, odds ratio; TT, total thyroidectomy; TIRADS, thyroid imaging reporting and data systems.

Multivariate analysis showed that the presence of hypothyroidism (OR = 3.230, 95% CI: 1.368–7.624, *P* = 0.007), CCND (OR = 2.196, 95% CI: 1.133–4.257, *P* = 0.02) and parathyroid in surgical specimen (OR = 5.547, 95% CI: 3.065–10.036, *P* < 0.0001) were independent predictors of transient hypoparathyroidism (Table 4). Among 78 patients who had nodal metastasis, the occurrence of transient hypoparathyroidism was significantly higher (*P* = 0.008) in those who had node size >3 cm (73.3%).

**Table 4:** Multivariate analysis for factors predicting transient hypoparathyroidism and hypocalcaemia^a^.

Predictor	OR	95% CI		*P*
		Lower	Upper	
Hypoparathyroidism				
Comorbidity – Hypothyroidism	3.230	1.368	7.624	0.007
CCND – Yes	2.196	1.133	4.257	0.020
Parathyroid gland in specimen – Yes	5.547	3.065	10.036	<0.0001
Hypocalcaemia				
Female – Gender	2.689	1.049	6.895	0.039
CCND – Yes	2.192	0.990	4.850	0.05
Parathyroid gland in specimen – Yes	1.067	0.367	8.438	0.004

^a^Logistic regression.

CCND, central compartment neck dissection.

Multivariate analysis showed that the female gender (OR = 2.689, 95% CI: 1.049–6.895, *P* = 0.039), presence of parathyroid in the surgical specimen (OR = 1.067, 95% CI: 0.367–8.438, *P* = 0.004) and CCND (OR = 2.192, 95% CI: 0.990–4.850, *P* = 0.05) were independent predictors for postoperative transient hypocalcaemia (Table 4).

Seven patients (2.3%) developed permanent hypoparathyroidism. It was noted that all were female, the majority were less than 40 years old (71.4%), and all except one had malignancy in final histopathology. TIRADS 5 thyroid nodule was noted in 43%, and 71.4% had a preoperative diagnosis of malignancy with FNAC. CCND was performed in 43%, and thyroiditis was noted in 71.4% of cases. POD 1 PTH levels of less than 10 pg/mL was noted in 71.4%, and 85.7% had signs and symptoms of hypocalcaemia. The majority (85.7%) required both calcium and vitamin D supplements immediately following surgery.

Receiver operating characteristics curve analysis was performed to assess the PTH cutoff that better predicts POD 1 hypocalcaemia. It was found that a PTH value <10 pg/mL as this cutoff had a better sensitivity (79.5% vs 74.1%) than a cutoff of <15 pg/mL (AUC) in predicting POD 1 hypocalcaemia.

## Discussion

Hypoparathyroidism and hypocalcaemia are frequent complications of TT, with the majority being transient (Puzziello *et al.* 2015). In our study, transient hypoparathyroidism was noted in 26.7% of patients, and transient hypocalcaemia occurred in 12.3%. In a study by Nair *et al.*, among 806 patients, they noted transient hypocalcaemia in 23.6% of patients (Nair *et al.* 2013). In a similar study by Falch *et al.* in 702 patients, the incidence of transient hypocalcaemia was noted in 22.8% of patients (Falch *et al.* 2018). However, the problem in reporting postoperative transient hypocalcaemia is the lack of a uniform definition, as the definitions are very inconsistent, ranging from a simple chemical lab reference range for serum calcium levels to the presence of clinical symptoms and parathormone serum levels at various time points. In our study, postoperative transient hypoparathyroidism was defined as POD 1 PTH less than 10 pg/mL. Postoperative transient hypocalcaemia was defined as a POD 1 serum calcium level of less than 8 mg/dL, based on evidence from a meta-analysis (Nagel *et al.* 2022).

CCND and inadvertent removal of the parathyroid gland during surgery, which was confirmed by histopathological examination, were independent predictors for both transient hypoparathyroidism and POD1 hypocalcaemia. In addition, hypothyroidism was an independent predictor of transient hypoparathyroidism, and female gender was identified as a risk factor for POD1 hypocalcaemia.

Variations in the anatomical locations and blood supply of the parathyroid glands, along with the difficulty in distinguishing parathyroid glands from other cervical tissues due to their small size and similar colour compared to the thyroid, fat and lymph nodes, as well as their fragile nature, set the stage for their functional derangement following surgical manipulation. The mechanisms that underlie hypocalcaemia are related to disruption of the parathyroid arterial supply, interference with venous drainage, mechanical injury and thermal or electrical injury, which could lead to the intentional or unintentional partial or complete removal of parathyroid tissue (Orloff *et al.* 2018).

Extensive thyroid surgery has been reported to markedly increase the incidence of both transient and permanent hypoparathyroidism when compared with less extensive thyroid procedures. Transient hypoparathyroidism may develop in patients with thyroid cancer who undergo CCND due to vascular damage or inadvertent parathyroidectomy during the procedure. The superior parathyroid glands are at a lower risk of injury. The inadvertent removal of inferior parathyroid glands is more common because most central neck lymph node metastases are generally located in the paratracheal and pre-tracheal areas, along with the variability of parathyroid gland anatomy. Patients with thyroid cancer who undergo CCND are more likely to suffer from hypoparathyroidism, as injury to the lower parathyroid gland is often unnoticed in CCND (Orloff *et al.* 2018). Careful dissection preserving the blood supply to parathyroids (especially superior parathyroids) may help reduce the incidence of hypoparathyroidism. When we perform an ipsilateral CCND, the risk of contralateral central neck lymph node metastasis must be weighed against the risk of hypoparathyroidism and disease status. Then, a decision is taken regarding whether to proceed with further nodal dissection (Orloff *et al.* 2018). A meta-analysis by Qin *et al.* identified that surgery in patients with malignant nodules was a significant risk factor for transient and permanent hypoparathyroidism (Qin *et al.* 2021).

In univariate analysis for temporary hypoparathyroidism, the presence of CCN on imaging, the presence of metastatic nodes in the specimen, and a pathological metastatic node size >3 cm were related to transient hypoparathyroidism, but they were excluded in multivariate analysis because of wide CIs. However, it is essential to note that the presence of significant lymph nodes on ultrasonography imaging or intraoperative findings that dictate CCND may increase the risk of transient hypoparathyroidism and transient hypocalcaemia. Also, this risk increases exponentially when the size of any node is more than 3 cm, which may be related to the burden of disease and, hence, the need for more extensive surgery.

Dissection along the capsule of the thyroid is required to safeguard the parathyroid. This technique is complicated by the presence of extrathyroidal extension, fibrosis or increased vascularity due to thyroiditis, as well as the surgeon's experience. Transient hypoparathyroidism is likely to occur when one or more parathyroid glands are excised inadvertently (Rajinikanth *et al.* 2009, Sitges-Serra *et al.* 2010). According to a meta-analysis by Qin *et al.*, the presence of a parathyroid gland in the histology specimen significantly increases the incidence of both transient and permanent hypoparathyroidism (Qin *et al.* 2021). In our study, 79 patients (26.3%) had parathyroid gland tissue identified in the histopathology specimen, of which 53.2% had transient hypoparathyroidism and 22.8% had transient hypocalcaemia. In their study, Sitges-Serra *et al.* found inadvertent parathyroid excision in 16.1% of 442 patients. However, they had more benign disease (77.6%), unlike in our cohort, where we had malignancies (80.3%) diagnosed (Sitges-Serra *et al.* 2010). Similar findings were reported by Rajinikanth *et al.,* where among 365 patients, inadvertent parathyroid excision was seen in 12.91%, with 58.9% of the study population having benign diagnosis (Rajinikanth *et al.* 2009). Of 300 patients, 205 (68.3%) had all parathyroid glands identified and preserved during surgery. Of 59 patients who underwent CCND, 39% had parathyroid glands in the specimen.

The presence of comorbidities like diabetes mellitus, hypertension and hypothyroidism as predictors of hypoparathyroidism has not been described in the literature. In multivariate analysis, we found that hypothyroidism (OR = 3.230, 95% CI: 1.368–7.624, *P* = 0.007) was an independent predictor of transient hypoparathyroidism. Although not statistically significant, it was noted that patients with hypothyroidism had a higher prevalence of thyroiditis (46.9% vs 35.1%) and a relatively higher nodal disease burden (37.5% vs 31.2%) in malignant disease. These factors combined will pose challenges during thyroid surgery and might have compromised the parathyroid blood supply.

Studies have found that post-thyroidectomy hypocalcaemia develops more frequently in women than in men, probably because of hormonal factors related to the perimenopausal period, vitamin D deficiency and osteoporosis (Erbil *et al.* 2009, Lang *et al.* 2016). Although the study by Sands *et al.* found no difference in the incidence of hypocalcaemia between premenopausal and postmenopausal women, both groups of women exhibited a higher incidence compared to males (Sands *et al.* 2011). In our study, the female gender had a higher risk of POD1 hypocalcaemia than the male gender (OR = 2.689, 95% CI: 1.049–6.895, *P* = 0.039) in multivariate analysis (Table 4). Edafe *et al.* found that the female sex had a higher risk of postoperative hypocalcaemia than male patients (Edafe *et al.* 2014). Another meta-analysis by Qin *et al.* also showed that the female sex (Qin *et al.* 2021) had a higher incidence of transient hypocalcaemia.

The value of PTH in predicting post-thyroidectomy hypocalcaemia has been investigated extensively and reported in the literature. PTH is the most essential biochemical indicator of calcium levels, which usually reflects the functioning of the parathyroid gland. We found a significant correlation (*r* = 0.5, *P*-value: 2.2e–16) between POD 1 serum PTH and POD 1 serum calcium levels (Fig. 3). Among patients who developed transient hypocalcaemia, 72.9% also had transient hypoparathyroidism. Therefore, PTH values, in conjunction with calcium values, patient symptoms and signs of hypocalcaemia, can be a valuable tool to manage patients in the postoperative period.

The predictors of hypocalcaemia and hypoparathyroidism vary slightly in our study. Low PTH remains the main driver behind low serum calcium, though the presence of pre-existing low calcium reserve, osteoporosis (especially in females), renal failure and malabsorption may play a role in low calcium levels despite a normal PTH level. We found that PTH levels had a better correlation than calcium levels with the symptoms and signs of hypocalcaemia, and there was only a moderate correlation between POD 1 PTH and calcium levels (Fig. 3). Hence, post-operative hypocalcaemia may be better managed by using PTH levels as the primary decision-making tool, with calcium levels and clinical findings serving as adjuncts in the decision-making process. The presence of hypocalcaemic symptoms and signs in a proportion of patients with normal calcium could be due to an acute drop in calcium from baseline, although this would not satisfy the definition of hypocalcaemia.

The prevalence of permanent hypoparathyroidism was 2.3% in our study population. It is important to identify this group because studies have noted that 15% of patients in this group will not receive adequate treatment. If not properly managed, they might need repeated hospitalisation or can go on to develop neuropsychiatric symptoms, seizures, cardiac complications, cataracts and might even be fatal (Díez *et al.* 2019).

In our cohort, there was a high incidence of malignancy (80.3%). The prevalence of malignancy was higher in young females in our study compared to the Western population. A cancer registry in India has shown that thyroid cancer is the second most common cancer in Indian women aged 15–39 years (Sathishkumar *et al.* 2022).

Multiple studies have shown conflicting results with regard to vitamin D and hypoparathyroidism (Barbier *et al.* 2022, Qi *et al.* 2022, Saibene *et al.* 2022, Zhang *et al.* 2023). A retrospective study by Zhang *et al.* on 242 thyroid malignancy patients noted no significant relation between vitamin D and hypoparathyroidism, but they noticed that patients who had vitamin D deficiency had a higher risk of PTH reduction when compared to preoperative levels (Zhang *et al.* 2023). Similarly, a study from our institution noted vitamin D deficiency in 53.3% of subjects who underwent thyroidectomy, and vitamin D deficiency did not significantly influence postoperative hypoparathyroidism. However, the study noted a statistically significant increased duration of temporary hypocalcaemia among subjects with vitamin D deficiency. This might be related to reduced calcium absorption in the intestine due to vitamin D deficiency (Cherian *et al.* 2016).

This study is one of the largest single-centre series to look at predictive factors of postoperative hypocalcaemia. The PTH cut-off used differs from other studies, as we found a level of 10 pg/mL to have better sensitivity than 15 pg/mL. Our study has shown clearly that predictors for low PTH and calcium differ, and this has to be taken into consideration when tailoring postoperative management. Surgical technique plays a significant role in reducing rates of postoperative hypocalcaemia, as evidenced by the fact that inadvertent parathyroidectomy remains an independent common predictor of both POD 1 low PTH and calcium levels. The disadvantages of this study are its retrospective design and a lack of data on preoperative vitamin D levels.

## Conclusion

Hypoparathyroidism and hypocalcaemia after thyroid surgery are common, and most of them are transient. Predictors differ for hypoparathyroidism and POD 1 hypocalcaemia. Postoperative hypocalcaemia may be better predicted and treated by using PTH as the primary decision-making tool, with calcium levels/clinical findings serving as an adjunct.

## Declaration of interest

There is no conflict of interest that could be perceived as prejudicing the impartiality of the research reported.

## Funding

This research did not receive any specific grant from any funding agency in the public, commercial or not-for-profit sector.

## Ethical approval and consent

This study obtained ethical approval from the Institutional Review Board (IRB min no. 15756(retro), dated 20.09.2023) Christian Medical College, Vellore. Written informed consent was waived due to the retrospective nature of the study.

## Author contribution statement

JJL: Data collection & analysis, Interpretation & Conclusion, Preparation of Manuscript. JR: Research & Study design, Interpretation & Conclusion, Review of Manuscript, Guide & Critical Revision. NR: Research & Study design, Data collection & analysis, Interpretation & Conclusion, Preparation of Manuscript. AAP: Data collection & analysis. RCM: Review of Manuscript, Guide & Critical Revision. AJT: Review of Manuscript, Guide & Critical Revision. RR: Review of Manuscript, Technical Support. TA: Review of Manuscript, Technical Support. SS: Review of Manuscript, Technical Support. RN: Data collection & analysis, Technical Support.

## References

[bib1] Barbier MP Mingote E Sforza N Morosán Allo Y Lotartaro M Serrano L Fossati MP Meroño T Faingold C Sedlinsky C, *et al.* 2022 Incidence and predictive factors of postoperative hypocalcaemia according to type of thyroid surgery in older adults. Endocrine 75 276–283. (10.1007/s12020-021-02840-9) 34350564

[bib2] Cherian AJ Ponraj S Gowri S M Ramakant P Paul TV Abraham DT & Paul MJ 2016 The role of vitamin D in post-thyroidectomy hypocalcemia: still an enigma. Surgery 159 532–538. (10.1016/j.surg.2015.08.014) 26365947

[bib3] Díez JJ Anda E Sastre J Pérez Corral B Álvarez-Escolá C Manjón L Paja M Sambo M Santiago Fernández P Blanco Carrera C, *et al.* 2019 Prevalence and risk factors for hypoparathyroidism following total thyroidectomy in Spain: a multicentric and nation-wide retrospective analysis. Endocrine 66 405–415. (10.1007/s12020-019-02014-8) 31317524

[bib4] Edafe O Antakia R Laskar N Uttley L & Balasubramanian SP 2014 Systematic review and meta-analysis of predictors of post-thyroidectomy hypocalcaemia. British Journal of Surgery 101 307–320. (10.1002/bjs.9384) 24402815

[bib5] Erbil Y Barbaros U Temel B Turkoglu U Işsever H Bozbora A Ozarmağan S & Tezelman S 2009 The impact of age, vitamin D(3) level, and incidental parathyroidectomy on postoperative hypocalcemia after total or near total thyroidectomy. American Journal of Surgery 197 439–446. (10.1016/j.amjsurg.2008.01.032) 19324110

[bib6] Falch C Hornig J Senne M Braun M Konigsrainer A Kirschniak A & Muller S 2018 Factors predicting hypocalcemia after total thyroidectomy – a retrospective cohort analysis. International Journal of Surgery 55 46–50. (10.1016/j.ijsu.2018.05.014) 29777882

[bib7] Kitahara CM & Sosa JA 2016 The changing incidence of thyroid cancer. Nature Reviews. Endocrinology 12 646–653. (10.1038/nrendo.2016.110) PMC10311569 27418023

[bib8] Lang BH-H Chan DTY & Chow FC-L 2016 Visualizing fewer parathyroid glands may be associated with lower hypoparathyroidism following total thyroidectomy. Langenbeck’s Archives of Surgery 401 231–238. (10.1007/s00423-016-1386-3) 26892668

[bib9] Nagel K Hendricks A Lenschow C Meir M Hahner S Fassnacht M Wiegering A Germer C-T & Schlegel N 2022 Definition and diagnosis of postsurgical hypoparathyroidism after thyroid surgery: meta-analysis. BJS Open 6 zrac102. (10.1093/bjsopen/zrac102) 36050906 PMC9437325

[bib10] Nair CG Babu MJC Menon R & Jacob P 2013 Hypocalcaemia following total thyroidectomy: an analysis of 806 patients. Indian Journal of Endocrinology and Metabolism 17 298–303. (10.4103/2230-8210.109718) 23776907 PMC3683209

[bib11] Orloff LA Wiseman SM Bernet VJ Fahey TJ Shaha AR Shindo ML Snyder SK Stack BC Sunwoo JB & Wang MB 2018 American Thyroid Association statement on postoperative hypoparathyroidism: diagnosis, prevention, and management in adults. Thyroid 28 830–841. (10.1089/thy.2017.0309) 29848235

[bib12] Promberger R Ott J Bures C Kober F Freissmuth M Seemann R & Hermann M 2014 Can a surgeon predict the risk of postoperative hypoparathyroidism during thyroid surgery? A prospective study on self-assessment by experts. American Journal of Surgery 208 13–20. (10.1016/j.amjsurg.2013.11.007) 24746378

[bib13] Puzziello A Gervasi R Orlando G Innaro N Vitale M & Sacco R 2015 Hypocalcaemia after total thyroidectomy: could intact parathyroid hormone be a predictive factor for transient postoperative hypocalcemia? Surgery 157 344–348. (10.1016/j.surg.2014.09.004) 25616948

[bib14] Qi Y Chai J Zhang L & Chen Y 2022 Preoperative vitamin D level is significantly associated with hypocalcemia after total thyroidectomy. BMC Musculoskeletal Disorders 23 1118. (10.1186/s12891-022-05977-4) 36550431 PMC9773437

[bib15] Qin Y Sun W Wang Z Dong W He L Zhang T & Zhang H 2021 A meta-analysis of risk factors for transient and permanent hypocalcemia after total thyroidectomy. Frontiers in Oncology 10 614089. (10.3389/fonc.2020.614089) 33718114 PMC7943836

[bib16] Rajinikanth J Paul MJ Abraham DT Ben Selvan CK & Nair A 2009 Surgical audit of inadvertent parathyroidectomy during total thyroidectomy: incidence, risk factors, and outcome. Medscape Journal of Medicine 11 29. (10.1111/j.1445-2197.2009.04916_23.x) 19295950 PMC2654678

[bib17] Saibene AM Rosso C Felisati G Pipolo C De Leo S Lozza P Cozzolino MG & De Pasquale L 2022 Can preoperative 25-hydroxyvitamin D levels predict transient hypocalcemia after total thyroidectomy? Updates in Surgery 74 309–316. (10.1007/s13304-021-01158-5) 34564834 PMC8827121

[bib18] Sands NB Payne RJ Côté V Hier MP Black MJ & Tamilia M 2011 Female gender as a risk factor for transient post-thyroidectomy hypocalcemia. Otolaryngology–Head and Neck Surgery 145 561–564. (10.1177/0194599811414511) 21750342

[bib19] Sathishkumar K Chaturvedi M Das P Stephen S & Mathur P 2022 Cancer incidence estimates for 2022 & projection for 2025: result from National Cancer Registry Programme, India. Indian Journal of Medical Research 156 598–607. (10.4103/ijmr.ijmr_1821_22) 36510887 PMC10231735

[bib20] Shoback DM Bilezikian JP Costa AG Dempster D Dralle H Khan AA Peacock M Raffaelli M Silva BC Thakker RV, *et al.* 2016 Presentation of hypoparathyroidism: etiologies and clinical features. Journal of Clinical Endocrinology and Metabolism 101 2300–2312. (10.1210/jc.2015-3909) 26943721

[bib21] Sitges-Serra A Ruiz S Girvent M Manjón H Dueñas JP & Sancho JJ 2010 Outcome of protracted hypoparathyroidism after total thyroidectomy. British Journal of Surgery 97 1687–1695. (10.1002/bjs.7219) 20730856

[bib22] Zhang L Li Z Zhang M Zou H Bai Y Liu Y Lv J Lv L Liu P Deng Z, *et al.* 2023 Advances in the molecular mechanism and targeted therapy of radioactive-iodine refractory differentiated thyroid cancer. Medical Oncology 40 258. (10.1007/s12032-023-02098-3) 37524925

